# Comparative Blood-Based Transcriptomic Profiles of Prostate Cancer Patients from South Africa and the USA: A Cross-Sectional Pilot Study

**DOI:** 10.7150/jca.126397

**Published:** 2026-01-14

**Authors:** Srinivas V Koduru, Mark Kidd, Ané Pieters, S E Nagel, Robert P Millar, Abdel B Halim

**Affiliations:** 1 Wren Laboratories LLC, Branford CT 06405, USA.; 2 Faculty of Health Sciences, University of Pretoria, Pretoria, South Africa; 3 Department of Integrative Biological Sciences, Institute of Infectious Diseases and Molecular Medicince, University of Cape Town.

## Abstract

Prostate cancer (PCa) is a major health problem worldwide with variable incidence, progression and outcomes depending on genetic, environmental and socio-economic factors. This study compares gene expression profiles in PCa patients from South Africa (RSA) and the United States (USA) using RNA sequencing in whole blood and pathway analyses. Whole blood samples were collected in Wren RNA stabilization tubes from RSA-PCa (*n* = 6), RSA-controls (*n* = 6), USA-PCa (*n* = 7) and USA-Controls (*n* = 11). RNA sequencing revealed 1,627 differentially expressed genes (DEGs) in RSA-PCa vs. RSA-controls, and 2,193 DEGs in USA-PCa vs. USA-Controls. Pathway analyses identified geographical region-specific variations; RSA-PCa had upregulated myeloid suppressor cell pathways and immunosuppressive markers while USA-PCa samples exhibited upregulated cytokine signaling and inflammatory pathways. Comparative analysis of healthy controls revealed 2,280 DEGs, which indicated significant differences in molecular profile of the geographic locations. qRT-PCR undertaken on 27 biomarkers related to PCa in whole blood (PROSTest) identified that 26 (96%) of the marker genes were commonly expressed. RNAseq and normalized PCR gene expression of these markers were well-correlated (r = 0.44, *p* = 0.0012, *n* = 30 pairs). The results of this study indicate that there are geographic differences in blood-based gene expression in both controls and individuals with PCa. Genes associated with a clinically validated molecular assay (PROSTest) were identified in both populations, but significant differences in gene expression relevant to tumor pathobiology were identified. These immune-associated signaling pathways suggest differences between these two cohorts in blood-based molecular architecture related to PCa. They also suggest the need to consider population-specific biomarkers to better understand this disease. Ultimately, optimizing blood-based molecular diagnostic and therapeutic approaches will require population-level studies.

## Introduction

Prostate cancer (PCa) is a significant public health concern and one of the most frequently diagnosed malignancies in men worldwide. It is the second most common cancer in men after lung cancer, and ~1.5 million new cases were diagnosed in 2022 1. The United States has the highest incidence; 313, 780 new cases were diagnosed in 2025, and the lifetime risk of being diagnosed with PCa is about 1 in 8 2.

PCa risk varies significantly across geographic, socioeconomic, and racial contexts. Predominant risk factors include age (from ~5% in < 30-year-old males to ~60% by age of 80), lifestyle (BMI and high alcohol intake, socioeconomic status), genetic burden (family history, inherited mutations in DNA damage and mismatch repair genes), and ethnicity 3-5. Men of African descent have a significantly elevated risk (2-3-fold) of having PCa compared to Caucasian, Hispanic/Latino and Asian men 6. In South Africa, PCa is the most commonly diagnosed cancer in African men, with a 60% increase in new cases reported between 2002 and 2018 7. This is attributed to increasing life expectancy, improved diagnostic capabilities, lifestyle changes, and environmental exposures 8. Disparities in healthcare access, such as unequal availability of diagnostic tools and treatment facilities, further exacerbate mortality rates in low-resource regions. These differences underscore the need for equitable diagnostic strategies and region-specific research to address the unique genetic and environmental factors influencing PCa 7.

Advances in next-generation sequencing (NGS) have transformed PCa research, offering insights into the molecular mechanisms of the disease 9. NGS facilitates comprehensive profiling of genetic and transcriptomic alterations, enabling the discovery of novel biomarkers for early detection, disease monitoring, and personalized treatment. Molecular testing, typically used at a tissue level, includes the development of several mRNA-based tools e.g., Oncotype Dx, that are now used in PCa management. Peripheral blood, however, provides an alternative testing compartment for RNA-based assays 10. CTCs and cfRNA are detectable in PCa blood 11, while platelets are a well-known source of tumor-derived mRNA 12. Whole blood-derived gene expression assays have been described 13, 14; different immune-related 6- and 9-gene or PCa tumor-associated 5-gene assays are available for research purposes 13-15. PROSTest is a clinically validated liquid biopsy that is a minimally invasive, mRNA-based diagnostic (27 target genes) with > 90% sensitivity that leverages molecular profiling of gene expression in whole blood samples 16, 17. While providing a source of “tumor signal”, peripheral blood also offers an opportunity to interrogate molecular information from circulating immune cells. This may provide insights into host and environmental responses.

In this study, we evaluated whether blood-based gene expression patterns in PCa between Caucasian US and Black South African populations was different. We posited than any differences could reflect variations in underlying genetic and environmental factors which are known to exist 8, 18, 19. Using RNA sequencing and liquid biopsy technologies, we compared transcriptomic signatures in two age- and tumor biology-matched sample sets as well as the clinically validated PROSTest PCR-based signature across these populations. The objective was to use these tools to provide insights into geographic/ethnic-specific disease pathobiology of PCa. We undertook this global approach but also focused on genes and pathways e.g., AR-signaling, known to differ between these ethnicities 20-22.

## Materials and Methods

**Clinical Samples:** Based on established protocols for RNAseq-based DEGs 23, we focused on a minimum of 6 biological samples from each of the 4 groups (PCa-USA, PCa-RSA, Con-USA, Con-RSA), that we wanted to evaluate. Whole blood samples were collected between 2020-24 [United States (USA-PCa: 7, USA-Control: 11), Republic of South Africa (RSA-PCa: 6, RSA-Control: 6)] in accordance with respective guidelines and regulations of both countries (United States: WIRB20191743 approved by the Western Institutional Review/WCG Board; South Africa: 389/2020 approved by the Research Ethnics Committee of the University of Pretoria, Reference number: GP202009 032 on the South African Health Research Database) and with the Helsinki declaration of 1975, as revised in 2013. All samples were collected from adult participants [USA median age: 61.5 yrs (USA-PCa: 66 yrs; USA-Controls: 57 yrs); RSA median age: 57.5 yrs (RSA-PCa: 63 yrs; RSA-Controls: 66 yrs)] and informed consent was obtained from all subjects in accordance with ethnical guidelines. All patients age, ethnicity, Gleason scores, disease status with various stages in Table 1.

**RNA isolation:** Total RNA was extracted from whole blood (at Wren Laboratories LLC, USA) using TRIzol (Thermofisher) and cleaning further by RNeasy Mini isolation kit (Qiagen) 24, 25. Briefly, 150 μl of whole blood was added to 750 μl of TRIzol LS, incubated for 5 minutes, vortexed with 200 μl of chloroform, and centrifuged at 13,000 rpm for 12 minutes. The aqueous phase was transferred to a new 2 ml sample tube and RNA isolation and purification was carried out using the QIACube MDx instrument (Qiagen) with RNeasy Mini Kit according to the manufacturer's instructions. RNA was eluted in 80 μl of nuclease-free water.

**RNA-seq quality control:** RNA quality was assessed using the A260/A280 and A260/A230 ratios measured by a NanoDrop spectrophotometer. RNA integrity was evaluated with an Agilent Bioanalyzer 2100, and samples with RIN values of 6.5 or greater were included in library preparation.

**RNA seq library prep:** mRNA was enriched from approximately 50 ng of total RNA using oligo-dT beads and fragmented by incubation at 94ºC in the presence of Mg^2+^ (Kapa mRNA Hyper Prep). First-strand cDNA synthesis was performed using random primers, followed by second-strand synthesis and A-tailing with dUTP to generate strand-specific sequencing libraries. Adapter ligation with 3' dTMP overhangs were ligated to library insert fragments. Library amplification amplifies fragments carrying the appropriate adapter sequences at both ends. Strands marked with dUTP were not amplified. Libraries were sequenced on an Illumina NovaSeq 6000 platform to generate 25 million reads per sample for further analysis (Yale University Core, USA).

**Flow cell preparation and sequencing:** Sample concentrations were normalized to 1.2 nM and loaded onto an Illumina NovaSeq flow cell at a density targeting 2 million passing filter clusters per sample. Sequencing was performed using 100 bp paired-end reads in accordance with Illumina protocols. A 10 bp unique dual index was used for sample identification. Positive control libraries (PhiX, 0.3%) were spiked in to monitor sequencing quality.

**Data extraction:** Signal intensities are converted to individual base calls during a run using the system's Real-Time Analysis software. Base calls were transferred from the machine's dedicated personal computer to the Yale High Performance Computing cluster via a 1 Gigabit network mount for downstream analysis. Primary analysis, including demultiplexing and genome alignment, was conducted using CASAVA 1.8.2 software.

**mRNA-seq data analysis:** FASTQ mRNA-Seq files were processed with Partek Flow® software, version 11.0.23.0918 (Partek, Inc., St. Louis, MO) on a Linux-based high performance computing system at Partek (40 cores, 503 GB RAM, 4TB disk space). The adapter was removed with CUTADAPT v4.2 and remapped into the human hg38 genome using the BWA aligner (v0.7.17) with default settings (minimum bases 24 read length) for read mapping 24, 26, 27. Gene annotation was performed using Ensembl v109, and transcript abundance was normalized with DESeq2 for statistical analysis with FDR correction. DEGs were identified using a threshold of *p* < 0.05 and fold-change > 2 24. Partek Flow is commercially available graphical interface web-based application, which allows the choice of variables and parameters for typical data analysis.

**Pathway analysis:** Pathway analysis was carried out using publicly available tools, including Metascape, Gene Ontology (GO), SRplots 28, 29 and g:Profiler. Functional analysis encompassed GO Molecular Functions, Biological Processes, Cellular Components, and pathway enrichment analysis were carried out on clusterProfiler and pathview 27, 30, 31. The results provided insights into molecular dysregulations associated with PCa across USA and RSA cohorts.

**Real-Time qPCR:** The above clinical samples RNA was converted into cDNA using High-Capacity cDNA Reverse Transcriptase Kit (ThermoFisher). Real-time PCR was carried out on pre-spotted TaqMan PCR primer plates [27 PROSTest genes (list of genes in **Table 3**)] using 200 ng of cDNA/well 16, 17. Positive controls were used on every spotted plate. Target transcript levels were normalized to *ALG9*, *TOX4* and *TPT1* and quantified using _ΔΔ_*C*_T_.

**Immune cell analysis:** Immune infiltration estimations for gene expression data was carried out using web-based tools including Immunedevconv with TIMER 2.0, CIBERSORT, quanTIseq, xCell, MCP-counter and EPIC algorithms 32-35.

**Statistics and data visualization:** All experiments were minimum of triplicates to validate reproducibility and *p*-value < 0.05 considered statistically significant (for RNA-seq, FDR corrected). Circos plots were used as they are a powerful visualization tool used to display complex relationships within large-scale RNA-seq data in circular layout with chromosome number and transcript position to identify gene expression hotspots 36.

## Results

*Clinical Data:* A total of 13 PCa (7 USA, 6 RSA) and 17 controls (11 USA, 6 RSA) were evaluated. There was no significant difference in median ages (USA-PCa: 60 yrs, RSA-PCa: 63.5 yrs, USA-Con: 57 yrs, RSA-Con: 64 yrs; Kruskal-Wallis test: *p* = 0.18). PCa patients were comparable in the two cohorts. The distribution of low grade (GG1-2) Gleason grade (USA-PCa: 5/7; RSA-PCa: 5/6) and stage I-II disease (USA-PCa: 5/7; RSA-PCa4/6) were similar in both cohorts. All patients were newly diagnosed (7/13 [54%]) or in an active surveillance program (6/13 [46%]). None were currently being treated.

### Cancer-related peripheral blood differences

**Figure 1:**
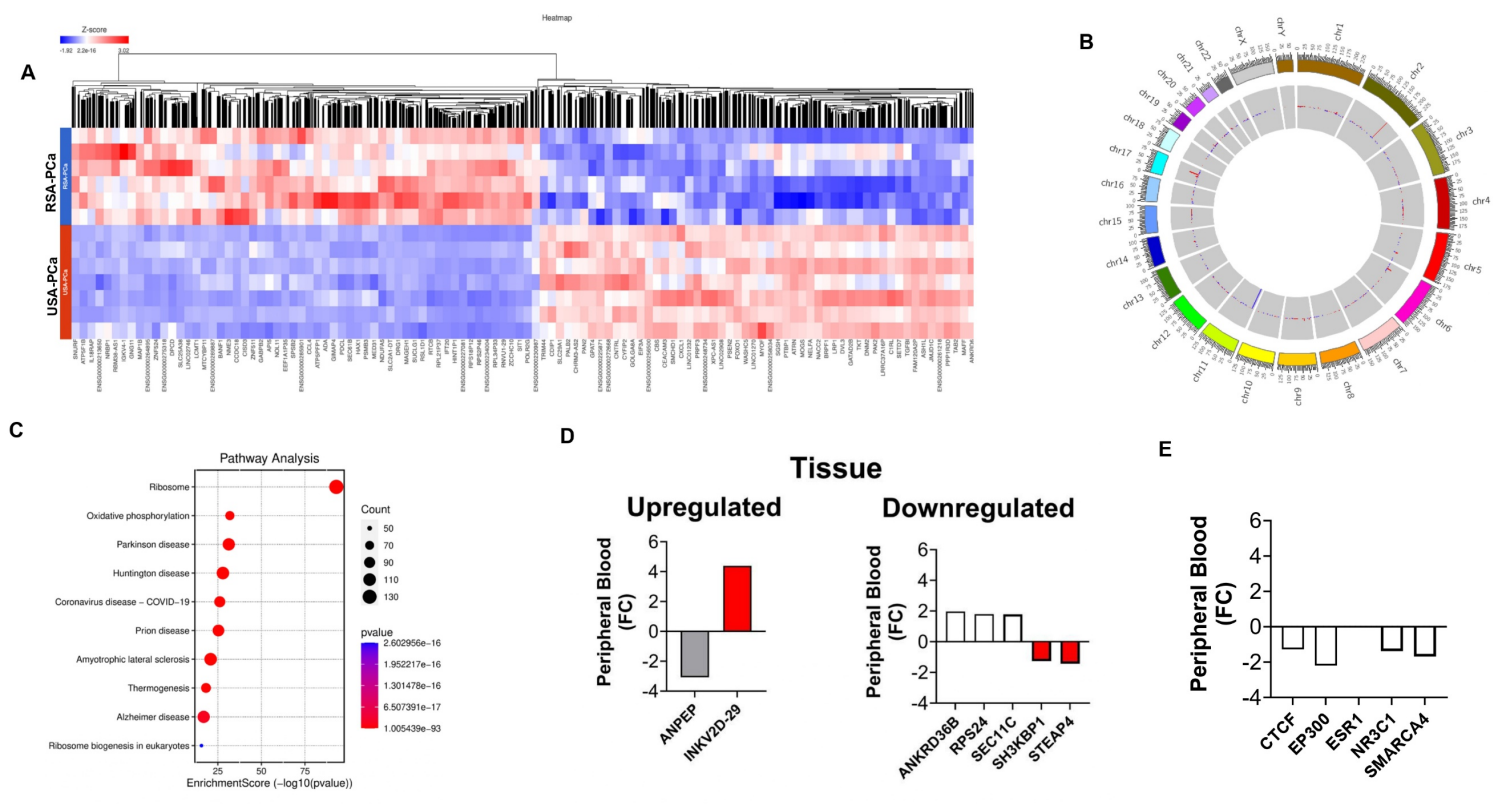
** Differential Gene Expression Analysis in Blood Samples from RSA and USA PCa Patients. 1A**. Hierarchal clustering (heatmap) of gene expression profiles in blood samples from RSA-PCa vs USA-PCa. The heatmap was generated on Partek Flow® software, version 11.0.23.0918 (www.partek.com). **1B**. Circos plots to visualize gene expression pattern in chromosomes between RSA and USA samples. Hotspots were identified in Chromosomes 1, 2, and 17. **1C**. Comparative visualization of pathways between RSA-PCa and USA-PCa groups highlights oxidative phosphorylation and disease-related pathways. **1D**. Peripheral blood expression of ethnic-associated tissue DEGs. The Immunoglobulin Kappa Variable 2D gene 29 was upregulated in blood, while both *SH3KBP1* and *STEAP4* were downregulated. These changes are consistent with tissue-based evaluations 20. **1E**. Four of ten previously identified master regulator transcription factors identified in PCa tumor tissues of Black men were downregulated in peripheral blood including *CTCF*, *EP300*, *NR3C1* and *SMARCA4*.

We initially compared RSA-PCa and USA-PCa sample sets to identify differences in blood-derived gene expression between subjects with PCa from these two different geographic regions. A total of 7,149 genes were identified to be differentially expressed with a FC > 2 (*p* < 0.05) (**Figure 1A**). The five most significant genes that were upregulated in RSA-PCa with the highest fold changes included *CERS6-AS1* (FC:238.1), *MTCO1P40* (FC:107.5), *ATP6V1G2-DDX39B* (FC:53.4), *MTND4P12* (FC:34.0), *RMRP* (FC:30.1), while the most down regulated genes were *GVINP1* (FC:-702.0), ENSG00000273217 (FC:-56.3; Rap Guanine Nucleotide Exchange Factor 6, associated with obesity and cholesterol metabolism), ENSG00000263244 (FC:-29.2; a novel transcript, 3' overlapping Chromosome 16 ORF72, associated with obesity and cholesterol metabolism), *LY75-CD302* (FC:-26.5) and *CXCL8* (FC:-10.6).

**Figure 1B** is a Circos plot that identified DEG hotspots in Chromosome 1, 2, and 17. None of these are associated with known genetic (GWAS) differences between Caucasians and Blacks 37 or were associated with the 27 PROSTest marker genes.

Pathway analyses (**Figure 1C**) identified the differences in Molecular adapter activity (signaling pathways), Molecular Carrier Activity (biomolecular transport), and Ubiquitin-Ubiquitin Ligase Activity. Biological processes including Cell Division, Mitochondrial Electron Transport: Growth Hormone Receptor Signaling and Fibroblast Proliferation were also noted to be different. These processes can all influence the tumor microenvironment, potentially impacting PCa development and progression.

PCa-associated genes known to be differentially expressed (between Caucasians and Blacks) at a tissue level 20 was then examined in the blood samples. We focused on the 10 most differentially expressed (up/down) identified by Kim *et al*. 20. Seven of the 20 were differentially expressed in blood (**Figure 1D**, **Supplementary Table 1**). Elevated expression (consistent with tumor tissue) was noted for *IGKV2-29* (FC: 4.39), a B cell/plasma cell-related gene associated with the tumor microenvironment. *STEAP4* (FC: -1.44) and *SH3KBP1* (FC: -1.26) were decreased. Both are associated with regulating PCa proliferation 38, 39. Kim *et al*., also identified 10 AR-associated master-regulators (at a tissue level) associated with PCa in Black subjects. Four were differentially expressed in blood; all were decreased in peripheral blood of Black RSA patients (**Figure 1E**, **Supplementary Table 1**).

Singh *et al*., 21 have previously defined a series of genes linked to racial differences in PCa. We examined these genes identifying upregulation of *CYP3A5* (FC: +1.78), genes involved in *HSD17B* (FC: +1.34 to +1.93) and a decreased in *BCL*-apoptosis genes (FC: -1.49 to - 1.91) (**Supplementary Figure 1 and Supplementary Table 2**).

Interrogation of the PCa receptor cistrome 22 has identified that androgen signaling may contribute to changes in lipid metabolism and the immune response including cytokine signaling. Androgen receptor expression is known to be differentially expressed (between Caucasians and Blacks) 20, 40. We evaluated families of genes for each of these pathways to evaluate whether the differences could be observed in peripheral blood (**Supplementary Table 3**). An evaluation of genes involved in lipid metabolism identified decreased expression of the majority of genes in Black PCa subjects but elevated expression of *FABP5* (a known PCa marker 41; FC: +2.15) was identified. *LPL* was also upregulated (FC: +2.24) in Black men. The majority of immune response genes had increased expression in Caucasian blood samples except for the *CCL* genes which was increased in Black men (FC: +1.43 to +3.68). *COX2* was downregulated in Black men (FC: -2.44).

### Differences between controls

**Figure 2:**
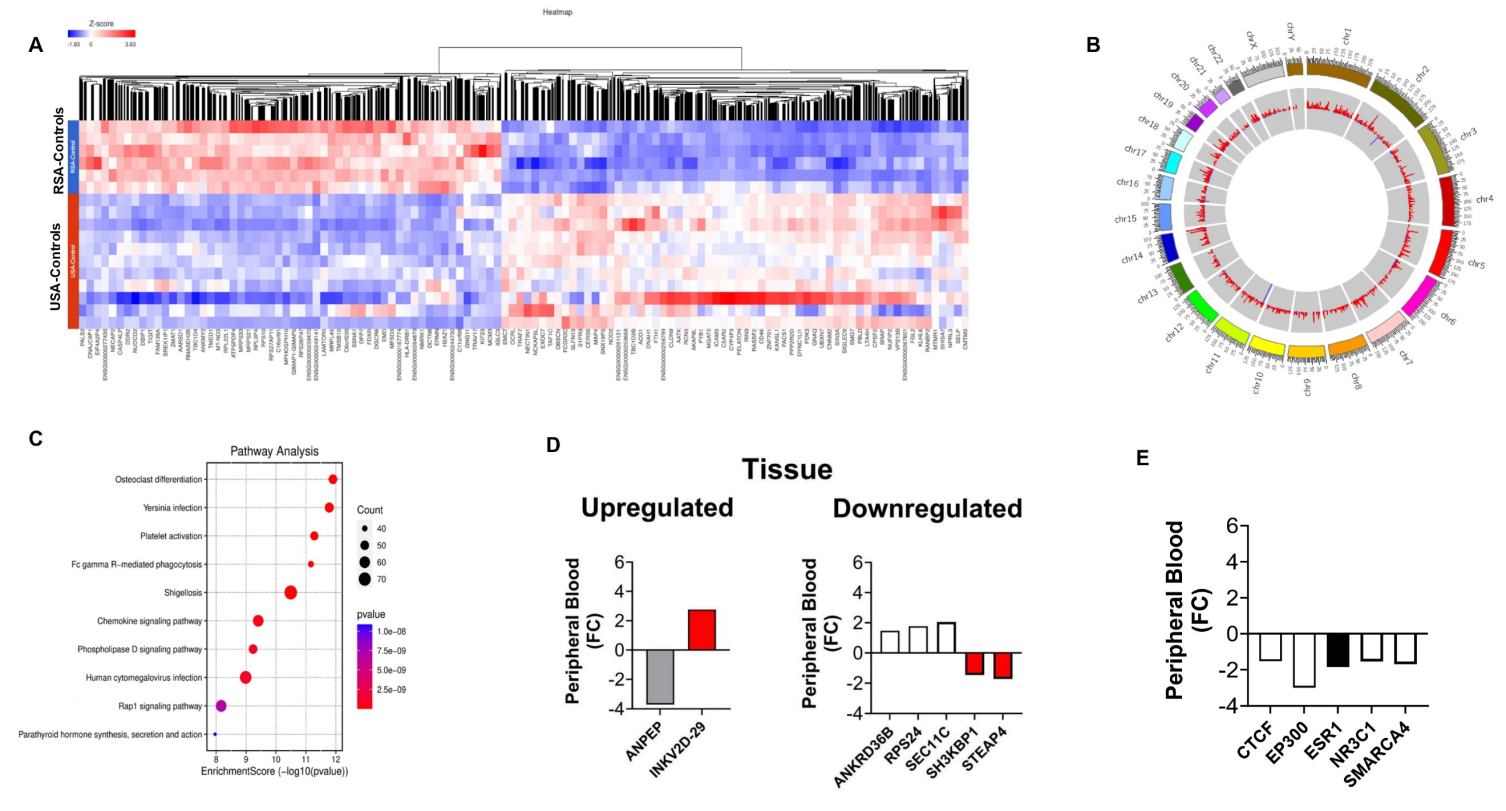
** Differential gene expression analysis in blood samples from RSA and USA controls. 2A**. Hierarchal clustering (heatmap) of gene expression profiles in blood samples from RSA-controls compared to USA controls. **2B**. Circos plots to visualize gene expression pattern in chromosomes between RSA and USA samples. Hotspots were noted in Chromosomes 1, 2, 5, 14, 17, and 19. **2C**. Comparative visualization of pathways between RSA-controls and USA-control groups highlights Protein Binding (Enzyme Regulatory Activity, mRNA Binding, and Zinc Ion Binding), Cell Division, and Cellular Responses to Insulin Stimulus. **2D**. Peripheral blood expression of ethnic-associated tissue DEGs. Changes in controls were similar to those in PCa blood samples (*see* **Figure 1D**). **2E**. The same master transcription factors as identified in PCa blood were downregulated in Black controls but *ESR1* was additionally identified to be decreased in expression.

We next compared gene expression in individuals in the control groups from RSA and the USA. Our analysis found 7,545 genes, with FC > 2 (*p* < 0.05) (**Figure 2A**). The five most significantly upregulated genes in RSA-Healthy controls were: *IGHG1* (FC: 10.3), *IGKV3-15* (FC: 8.6), *IFI27* (FC: 8.5), *MTCO1P40* (FC: 6.8) and *RMRP* (FC: 6.6) and the five most downregulated genes included *GVINP1* (FC: -574.1), *LY75-CD302* (FC: -313.4), ENSG00000263244 (FC: -109.8; a novel transcript, 3' overlapping Chromosome 16 ORF72, associated with obesity and cholesterol metabolism [also downregulated in RSA-PCa vs. USA-PCa]), ENSG00000272410 (FC: -20.0; a novel, uncharacterized protein associated with BMI) and *HTATSF1P2* (FC: -17.0).

**Figure 2B** informs differences at the “healthy” control level and identify those differences that occur at baseline (in the absence of disease) with hotspots noted in Chromosome 1, 2, 5, 14, 17, and 19. These differences may reflect genetic, environmental or lifestyle differences that are associated with the RSA and USA populations.

Pathway analysis (**Figure 2C**) identified the following GO molecular functions including: Protein Binding (Enzyme Regulatory Activity, mRNA Binding, and Zinc Ion Binding). Cell Division, Microtubule Nucleation, Cell Polarity and Cellular Response to Insulin Stimulus were also identified (**Supplementary Figure 2**).

We next examined whether the tumor-associated gene expression differences 20, racial disparity genes 21 and AR-signaling gene differences 22 were also evident in controls. Levels and differences were similar as noted for PCa blood (*see* **Figure 2D** and **2E**, and **Supplementary Tables 1-3**). The AR-master regulator associated transcription factor *ESR1* was decreased in Black men (FC: -1.85). In the immune profile, *IL15* and *IL23* were upregulated (FC: +1.34 to +1.98).

### Ethnic differences (PCa versus controls)

Given the differences between cancers (RSA vs. USA) and between controls from the two countries, our next approach was to compare and evaluate DEGs between cancers and control blood samples from the same country. A total of 16,533 transcripts were commonly detected (**Supplementary Figure 3A**). After statistical filtering (FC > 2, *p* < 0.05), we identified that ~10% of genes were commonly expressed (**Supplementary Figure 3B**).

*RSA-PCa vs Controls:* We identified 1,627 genes that were differentially expressed in PCa and control subjects with statistical significance (*p*-value < 0.05) and a fold change (FC) of > 2 (**Supplementary Figure 4A-D**). The five most differentially expressed genes include: ENSG00000270149 (FC: 7.2; a novel protein-coding transcript: TSTD1-F11R read-through linked to cholesterol metabolism), *HBZ* (FC: 5.9), ENSG00000288894 (FC: 5.6; a novel protein-coding transcript linked to cardiac and lipid metabolism), *CHIT1* (FC: 5.05), ENSG00000263798 (FC: 4.9; a lnc RNA associated with cholesterol metabolism). The five most down regulated genes in RSA-PCa blood included ENSG00000287510 (FC: -10.2; lncRNA antisense to the *LAPTM5* gene and related to immune function), *AGBL3* (FC:-4.16), ENSG00000228748 (FC:-4.1; a lncRNA, associated with cardiovascular function), ENSG00000290680 (FC:-3.8; linked to BMI and obesity) and LINC02470 (FC:-3.8; a lncRNA that regulates bladder tumorigenicity). Circos identified hotspots in Chromosome 1, 15, 16 and 17.

The significant top five pathways were identified as ribosome biogenesis in eukaryotes, mitophagy, spliceosome, ribosomal related genes and hematologic cell lineages. The results indicate an increased activity or sensitivity of hematological cells in RSA-PCa. This could be a sign of an immune activity or modulation or other immune responses that may be associated with tumor biology in this ethnic group.

*USA-PCa vs Controls:* In this cohort, we identified 2,193 genes with significantly different expression when compared to controls groups, with a FC > 2 and a *p*-value < 0.05 (**Supplementary Figure 5A-D**). Of the genes that were upregulated in USA-PCa, the five most differentially expressed were: *G0S2* (FC: 10.4), *EGR1* (FC: 8.4), ENSG00000248993 (FC: 6.8; a novel MHC Class II Beta Chain N-Terminal Domain-Containing Protein associated with obesity), ENSG00000279753 (FC: 5.8; an uncharacterized gene associated with cholesterol metabolism) and *OSM* (FC: 4.4). The five most down regulated genes were: ENSG00000287510 (FC: -59.5; lncRNA antisense to the *LAPTM5* gene and related to immune function), *C4BPA* (FC: -12.7), *MTND4P12* (FC: -6.4), ENSG00000280205 (FC: -6.0; an uncharacterized gene related to height) and *AIRN* (FC: -5.5). Circos identified hotspots in Chromosomes 1, 5, 6, 15 and 19.

Pathway analysis identified three major pathways (protein binding, microRNA binding, and ribonucleoprotein complex binding). When the focus was on biological processes, four major areas were discerned, these included cellular localization, cell cycle, cellular response to stress and apoptosis. These pathways differ from those in the RSA samples (RSA-PCa vs. RSA-controls) identifying some intriguing ethnic differences in blood-derived cell activity in PCa.

### Immune cell landscape analysis in USA and RSA populations

**Figure 3:**
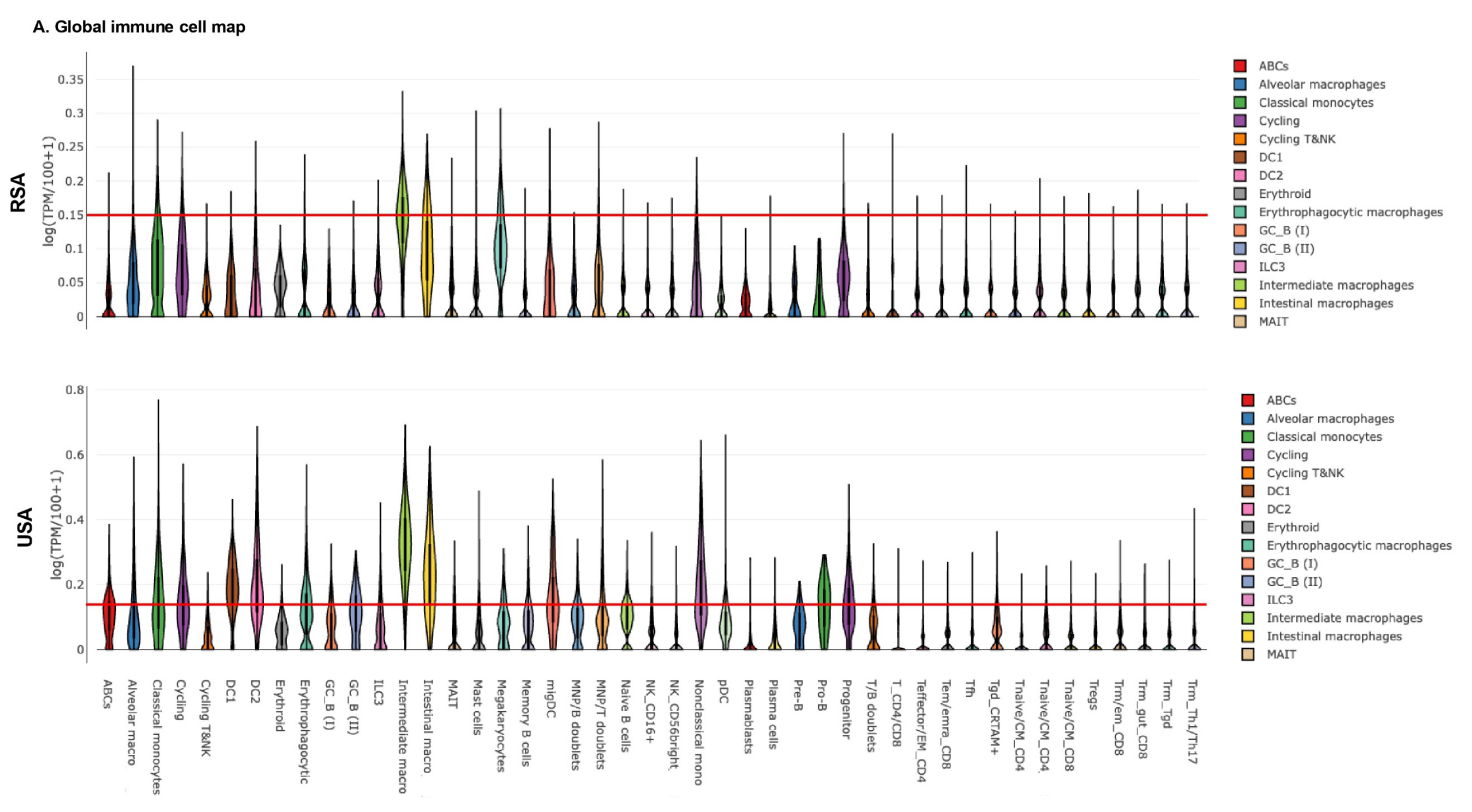

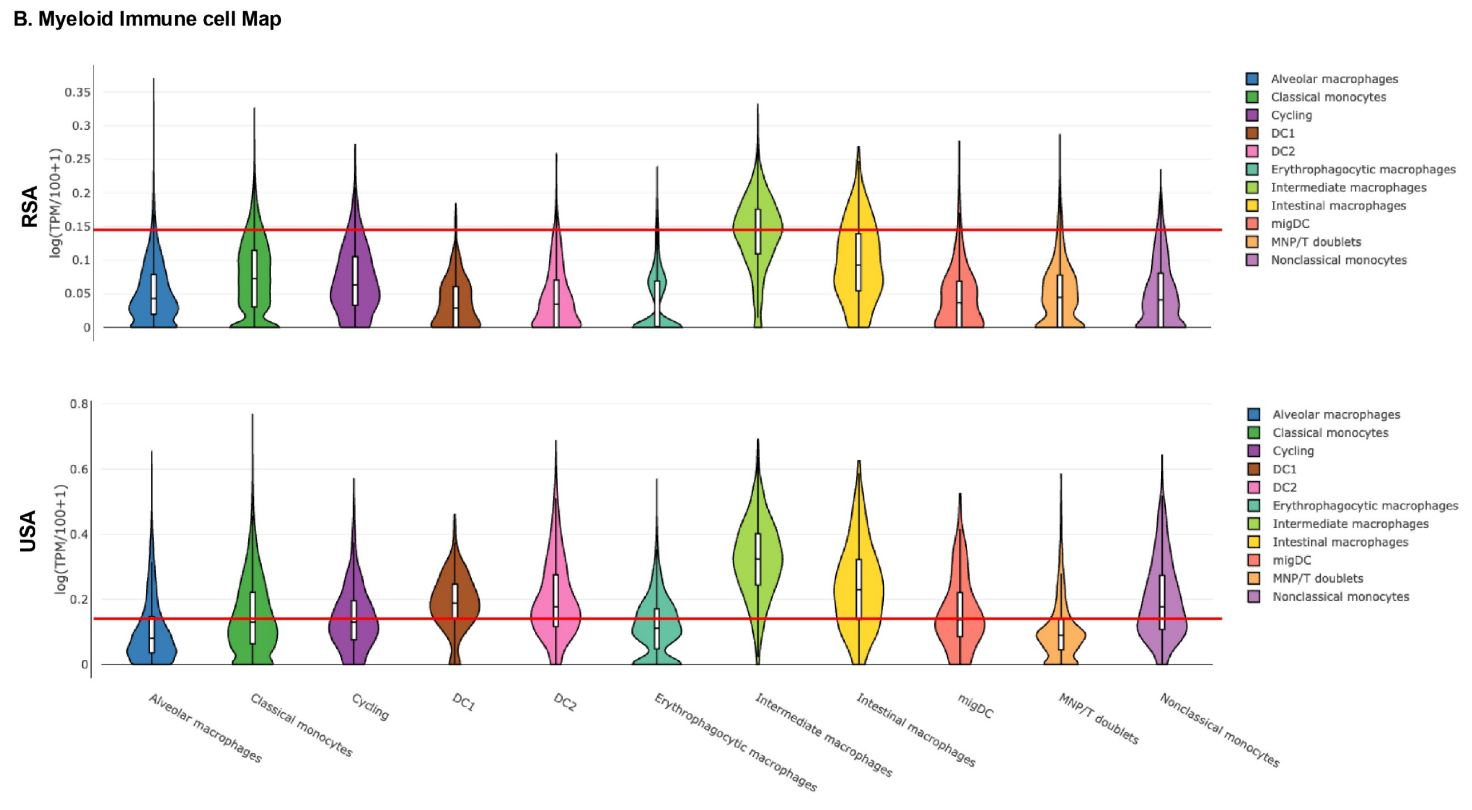
** Exploring global immune cell maps in RSA and USA populations. 3A.** Immune cell map analysis in RSA-PCa vs RSA-Controls (top) and USA-PCa vs USA-Controls(bottom) based on gene expression. Gene expression associated with various cell types and their log TPM values are included. The red lines are set at 0.15 as the scales are different. This provides for a comparison between the two groups which identifies over-expression of specific cell types including dendritic cells, macrophages and B cell types in Caucasian PCa peripheral blood.** 3B.** Myeloid immune cell map (top: RSA-PCa vs controls; bottom: USA-PCa vs. controls) of gene expression. The red lines are set at the same value (0.15) for ease of comparison. Dendritic cells, monocytes, and macrophage cell types are increased in Caucasian samples.

Given these findings, we next examined the peripheral blood immune landscape to better understand the population-based differences in the immune cell gene expression. For this, we performed global and myeloid immune cell analysis in RSA and USA cohorts based on DEGs. **Figure 3A-B** demonstrates global immune cell identification and variations in the PCa patients and the controls from RSA and the USA. We observed prominent differences in global immune cell landscape between these two populations. In general, the majority of the cell lineages were higher expressed in USA population (especially, ABCs, DC1, DC2, Erythrophagocytic Macrophages, Memory B cells and Naive B cells) compared to RSA (**Figure 3A-B**). The myeloid immune cell landscape using Myeloid Immune Cell mapping confirmed these differences (Macrophages, monocytes and dendritic cell types, **Figure 3C-D**).

**Table 2** includes a summary of the molecular pathway differences across RSA and USA Cohorts. These range from hematopoietic cell development to differences in lipid biosynthesis and inflammatory responses and immune cell populations.

### Evaluation of PROSTest genes in RNA-seq and qPCR

Lastly, we evaluated the expression of the 27 PROSTest genes (**Table 3**), which serves as established biomarkers of PCa identified in earlier studies at Wren Laboratories, in whole blood samples 16. This was undertaken to evaluate whether this tissue-derived gene signature was detectable in blood and whether there were any significant differences in expression of the marker genes.

**Figure 4:**
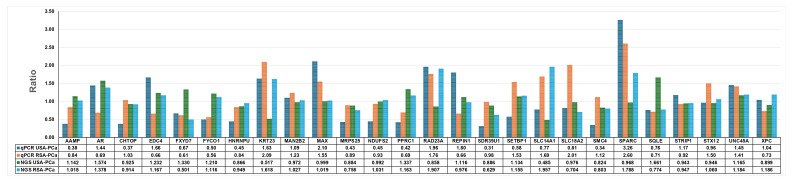
** Identification of PROSTest gene signature in RNA-seq data and comparison of qPCR ratios in RSA and USA clinical samples.** RNA-seq data identified 26 of 27 PROSTest genes and validated these genes via qPCR on same clinical samples that were sequenced. We observed all 26 genes via qPCR and bar graphs were prepared based on ratios in USA and RSA samples.

We employed two complementary methods, qPCR and RNA-seq. One of the 27 marker genes, *HPN* (hepsin), was not detected in the RNA-seq data and was excluded from analysis. Both qPCR and RNA-seq gave similar results for 26 PROSTest genes in the clinical samples (Pearson correlation: r=0.44, *p*=0.0012, *n*=30 pairs). This identified that these biomarkers are well and reproducibly detected irrespective of the method used (**Figure 4**, **Table 3**). The 26 PROSTest gene signature, was highly detectable in both populations with 100% sensitivity for differentiating PCa from healthy subjects (PROSTest genes and their RNA quantifications are listed in **Table 3**).

## Discussion

The research pilot study examined gene expression patterns in peripheral whole blood samples collected from PCa patients and healthy donors from South Africa and the United States. The cohorts were chosen to meet appropriate standards for RNAseq studies (a minimum of 6 samples/cohort 23), had similar ages (median ~60 years), were not being treated (either newly diagnosed or in active surveillance) and had similar Gleason grades and stages. This allowed us to minimize potential confounders and focus on evaluating differences in biology related to ethnicity and the environment.

RNA-seq data in these patients with localized, non-advanced tumors identified differential molecular variations between the two populations in terms of global gene expression patterns, pathway enrichment analysis and especially cell types involved in immune responses. These findings highlight some of the differences that genetic, environmental and socio-economic factors may have on PCa biology. They also suggest population-specific mechanisms that may be related or associated with disease (summarized in **Table 3**). We noted that PROSTest gene expression was similar between US and RSA PCa.

Authors have evaluated tumor samples for ethnic-associated gene and molecular pathway differences between Caucasia and Black PCa populations 20-22. Black men show increased activity of inflammation, steroid hormone responses, and cancer progression-related pathways. Ethnic-based androgen and androgen receptor (AR) pathway differences are known to occur. We evaluated differences at a global level and then focused on some of the previously identified genes and pathways known to differ.

At a global level, increased expression of genes in blood that were involved in biosynthesis of ribosomes, splicing of RNA, and hematopoietic cell lineages in the RSA cohort compared to US patients was identified. These pathways have been extensively studied in solid tumors. Alterations in ribosomal biogenesis and splicing (at a tissue level) are known to be correlated with tumor development including aggressiveness and metastasis 42, 43. PCa blood samples showed enhanced activity of pathways involved endoplasmic reticulum protein (ER) export stress which may be a key player in supporting tumor growth and treatment resistance 44. The oxidative phosphorylation and disease-specific pathways were also distinguished as different between the RSA and USA groups. These findings accord with research work showing geographical and genetic heterogeneity in the function of the mitochondria and its relationship to cancer metabolism 45, 46. An ER stress response leads to tumor cell activation which produces exosomes that contain UPR proteins which modify macrophage function and suppress T-cell activity 47. Our data demonstrates that elevated ER stress/export pathways serve as biomarkers which reveal proteostatic stress caused by tumors while linking protein folding needs to cancer progression levels.

We also observed striking differences in the baseline gene expressions between the RSA-Controls and USA-Controls further highlighting the importance of population variation. RSA controls demonstrated over-representation of cell cycle and mRNA binding pathways whereas USA controls showed enrichment in insulin response signaling. Such divergence in baseline expression indicates the differences in the basic molecular activity which could be due to different genetic background and environmental factors of the two populations. For example, populations with higher prevalence of metabolic syndrome or obesity (USA) may demonstrate enhanced insulin signaling, while subjects of African genetic heritage (RSA) may preferentially express proliferative and translational machinery activation 48-50. These baseline variations are similar to previously published large scale studies demonstrating significant ethnic variability in transcriptomes 51-54.

When we focused on specific genes and pathway differences noted at a tumor tissue level 20-22, we identified some intriguing overlaps between tissue-based expression and what was detectable in peripheral blood. Our study identified several tissue-associated differences were recapitulated in peripheral blood. For example, *FABP5* was over-expressed in RSA-PCa blood. This co-ordinates lipid signaling to promote PCa metastasis 55. Lipoprotein Lipase (*LPL*) is also linked to PCa by facilitating the uptake of fatty acids, which fuel tumor growth and proliferation 56. This gene similarly was upregulated in RSA blood compared to Caucasian blood. The NF-κB pathway was also upregulated in Black subjects; this controls progression of PCa to androgen-independent growth 57. Several genes transcribing interleukins were similarly upregulated in RSA-PCa samples. *IL32* has a complex role in PCas, acting as a pro-tumor factor in metastatic disease by recruiting macrophages that promote tumor growth and metastasis through the CTSZ-TRA2A/IL-32/ITGA5 axis 58. The *IL7R* (Interleukin-7 Receptor) was also upregulated and is implicated in PCa progression and potential therapeutic resistance 59. Chemokine signaling plays a significant role in PCa progression, metastasis, and the tumor microenvironment by recruiting immune cells and promoting tumor cell survival and growth. CCL4 signaling promotes tumorigenesis, invasion, and recurrence by recruiting macrophages and influencing the tumor microenvironment 60, while CCL5 promotes tumor growth, metastasis, angiogenesis (new blood vessel formation), drug resistance, and the self-renewal of cancer stem cells through its receptor CCR5 60. Both genes were upregulated in Black subjects the expression and role of Suppressor of Cytokine Signaling (SOCS) proteins are complex and can be tumor-promoting or tumor-suppressive depending on the specific SOCS protein and cellular context 61. Genes encoding proteins in this signaling pathway were upregulated too. Signals produced by tumors may affect blood cell development through mechanisms that lead to increased myeloid-derived suppressor cell (MDSC) numbers and altered lymphoid cell distribution 62, 63. The process of immunologic reprogramming is a key factor that drives cancer development and determines treatment outcomes. The immune-associated differences in peripheral blood suggest some fundamental differences in hematopoietic pathways between Caucasian and Black subjects; this may potentially impact on PCa development and progression.

**PROSTest Biomarker Validation:** The PROSTest biomarker panel containing 26 genes showed consistent results between RSA and USA cohorts identifying that these genes are reliable and stable biomarkers across these two populations. Our results also reveal the necessity of global population validation since molecular differences do occur in blood-based markers. Previous studies have highlighted the issues in the biomarker validation across the various populations with an emphasis on the impact of genetics, environment and socio-economic factors on biomarker performance 64-67. qPCR is a sensitive method that allows for the quantification of specific target genes, while RNA-seq offers the ability to confirm the presence and quantify the levels of expression of these genes in a global transcriptome setting 68.

**Geographical and Socio-Economic Considerations:** The differences in molecular signatures and the immune response DEGs (which likely translates to immune sub-population differences) between the RSA and USA cohorts are probably due to a genetically determined response to environmental exposures modulated by socio-economic factors. The fact that PCa has been noted to have different presentations in different geographical locations and ethnic groups has been well-established 69. This has been shown in the androgen receptor signaling, metabolic pathways and the immune response of the cancer varies between populations 70-75. Other factors that include diet (obesity, cholesterol metabolism, cardiovascular disease), infections and exposure to infectious agents and access to health care may also play a role in modulating gene expression and immunodynamics 76-80.

Our pilot study does have some limitations. The sample sizes are relatively small and uneven although we have attempted to meet requirements for RNAseq studies. One additional limitation was recruiting diverse ethnic groups, including RSA Caucasian and USA Black patients, to participate in the pilot study. Large-scale studies could further identify differences between these two groups (USA vs. RSA) and diverse racial pools, thereby enhancing generalizability and broader applicability. There may be a potential confounding by age, comorbidities, clinical stage distribution or sample handling differences but we have attempted to minimize these as far as possible. While no functional experiments, e.g., interleukin measurements in blood, were undertaken to confirm transcriptomic observations, these are being undertaken as part of a separate study. The qPCR vs. RNAseq expression of PROSTest genes, however, is a strength, that identifies the validity of the approach we used.

## Conclusion

The present pilot study highlights the need to perform population-specific analysis in any blood-based molecular research in order to develop biomarker and therapeutic strategies. This study also demonstrates the heterogeneity of immunological and cellular pathways among two different geographic (and ethnic) populations highlighting the need to consider these differences when evaluating PCa pathobiology. For instance, RSA-PCa samples were found to have characteristics suggestive of an impaired antigen presentation which may impact the effectiveness of the immune system in identifying and eradicating cancerous cells. Conversely, USA-PCa samples displayed an inflammatory immune signature suggesting an overall immune activation (monocyte, dendritic cells and macrophage DEGs). These suggest that blood samples from PCa are associated with different immune profiles, depending on the population. Such differences should be considered in studies as well as in clinical practice. In biomarker development, inclusion of subjects from different countries should be a principal focus in future genomic studies. Increasing the participation of different populations will help to increase the transferability of biomarkers and the interventions thus making them relevant to a wider population. Such inclusivity is especially important for the development of precision medicine which aims at providing individualized treatment based on the patient's genetic background as well as socio-economic environment.

### Clinical Implications

The determination of population attributable gene expression and immune signatures is important in PCa diagnosis and management. The alterations suggest different immune:cancer relationships that may have an impact on presentation, development and treatment of PCa. The confirmation of the PROSTest genes in both ethnic populations identifies that this non-invasive liquid biopsy-based diagnostic is a viable and effective tool for PCa.

## Supplementary Material

Supplementary figures and tables.

## Figures and Tables

**Table 1:** Clinical information of recruited patients

Patient No	Age	Diagnosis	Ethnicity	Status	Stage at Diagnosis	Gleason Score	Grade at Diagnosis	Staging (T)	Staging (N)	Staging (M)
USA-P1	76	PCa	C	ND	IC	7A	GG2	cT1c	NX	N/A
USA-P2	68	PCa	C	ND	IIB	7B	GG3	cT2b	cN0	M0
USA-P3	60	PCa	C	ND	IC	7B	GG3	cT1c	NX	N/A
USA-P4	58	PCa	C	AS	I	6	GG1	cT1	cN0	cM0
USA-P5	64	PCa	C	AS	I	6	GG1	pT1c3	N0	M0
USA-P6	57	PCa	C	ND	N/A	7A	GG2	pT3a	pN0	N/A
USA-P7	44	PCa	C	ND	N/A	7A	GG2	pT2	N0	N/A
RSA-P1	60	PCa	B	ND	T2A	6	GG1	pT2	N0	N/A
RSA-P2	68	PCa	B	ND	T1C	6	GG1	pT2	N0	N/A
RSA-P3	61	PCa	B	AS	T2C	7A	GG2	pT3a	pN0	N/A
RSA-P4	58	PCa	B	AS	T1C	6	GG1	cT1	cN1	cM0
RSA-P5	66	PCa	B	AS	T4	7A	GG2	pT3a	pN0	N/A
RSA-P6	66	PCa	B	AS	T4	8	G4	pT3a	pN0	N/A
USA-Healthy1	52	Control	C							
USA-Healthy2	57	Control	C							
USA-Healthy3	56	Control	C							
USA-Healthy4	54	Control	C							
USA-Healthy5	65	Control	C							
USA-Healthy6	58	Control	C							
USA-Healthy7	64	Control	C							
USA-Healthy8	54	Control	C							
USA-Healthy9	78	Control	C							
USA-Healthy10	45	Control	C							
USA-Healthy11	63	Control	C							
RSA-Healthy1	83	Control	B							
RSA-Healthy2	60	Control	B							
RSA-Healthy3	64	Control	B							
RSA-Healthy4	64	Control	B							
RSA-Healthy5	57	Control	B							
RSA-Healthy6	83	Control	B							

GG = Gleason Grade, ND = no data, C = Caucasian, B = Black, AS = Active Surveillance; ND = Newly Diagnosed, N/A = not available.

**Table 2:** Summary of molecular pathway differences and biomarker validation across RSA and USA cohorts

Category	RSA-PCa	USA-PCa	RSA-Controls	USA-Controls	Clinical/Translational Implications
Hematopoietic Lineages	↑ Enrichment of hematopoietic cell pathways (systemic immune modulation)	-	-	-	Suggests tumor-driven immune reprogramming detectable in blood.
Immune Gene Expression	↑ CCL genes ↑ *IL7R*, *IL18RAP*, *IL32*	↑ Multiple interleukins and NLRP genes	↑ IL*15*, *IL23A*, *IL32*	Similar but less pronounced as USA-PCa	Suggests interleukin gene expression differences between ethnic groups
Ribosome Biogenesis & RNA Splicing	↑ Increased expression of ribosomal genes and splicing machinery	Similar but less pronounced	-	-	Onco-ribosomes and aberrant splicing promote tumor aggressiveness; blood-based detection may allow early stratification of high-risk patients.
ER Stress & Protein Export	↑ Elevated ER stress and unfolded protein response (UPR) pathways	-	-	-	Links proteostasis stress to tumor growth and therapy resistance; potential therapeutic target.
Oxidative Phosphorylation (OXPHOS)	Distinctive differences vs. USA cohort	Distinctive differences vs. RSA cohort	-	-	Reflects population-specific mitochondrial metabolism; supports heterogeneity in cancer energetics and treatment response.
Baseline Molecular Signatures	-	-	↑ Cell cycle, mRNA binding pathways	↑ Insulin response pathways	Highlights genetic/environmental influences; critical for biomarker panel calibration across populations.
Fatty Acid Metabolism	Down-regulated compared to USA except for ↑ *FABP5* and *LPL*	Upregulated	Similar but less pronounced as RSA-PCa	Similar but less pronounced as USA-PCa	Metabolic differences between ethnic groups
Testosterone and Estrogen regulation	↑ *CYP3A5* and *HDS17B* family of genes	-	↑ *HDS17B* family of genes	-	Ethnic differences in Androgen regulation
PROSTest Validation	26-gene panel validated; consistent RNAseq-qPCR correlation	26-gene panel validated; consistent RNAseq-qPCR correlation	-	-	Robust across populations, but necessitates broader validation to ensure global utility.
Geographic & Socio-Economic Factors	Greater influence of genetic ancestry, environment, and infection exposure	Greater influence of lifestyle (diet, obesity, insulin signaling) and healthcare access	-	-	Socio-economic and geographic context strongly modulate molecular signatures; must be integrated into biomarker development strategies.

**Table 3:** PROSTest genes identified in RNA-seq data in USA and RSA patients.

	RNA Sequencing											
	USA-PCa vs USA-Controls			RSA-PCa vs RSA-Controls			USA-PCa vs RSA-PCa			USA-Controls vs RSA-Controls		
Gene name	P-value	FDR	Ratio	P-value	FDR	Ratio	P-value	FDR	Ratio	P-value	FDR	Ratio
**AAMP**	0.161	0.608	1.142	0.876	0.983	1.018	0.336	0.499	1.078	0.763	0.847	0.966
**AR**	0.110	0.533	1.574	0.376	0.843	1.378	0.725	0.825	1.128	0.981	0.990	0.993
**CHTOP**	0.334	0.755	0.925	0.262	0.789	0.914	0.186	0.328	0.935	0.439	0.583	0.928
**EDC4**	*0.010*	0.213	1.232	0.265	0.791	1.167	*0.000*	0.000	1.497	*0.002*	0.009	1.425
**FXYD7**	0.241	0.689	1.330	0.087	0.613	0.501	0.313	0.474	1.398	0.024	0.066	0.523
**FYCO1**	*0.013*	0.244	1.210	0.419	0.869	1.116	*0.000*	0.000	1.432	*0.015*	0.044	1.329
**HNRNPU**	*0.035*	0.354	0.866	0.530	0.908	0.949	*0.000*	0.001	1.187	*0.002*	0.007	1.308
**KRT23**	*0.008*	0.202	0.517	*0.033*	0.471	1.618	*0.033*	0.088	0.663	*0.008*	0.027	2.098
**MAN2B2**	0.779	0.946	0.972	0.858	0.981	1.027	*0.000*	0.000	1.709	*0.000*	0.000	1.819
**MAX**	0.990	0.997	0.999	0.869	0.982	1.019	0.889	0.933	1.015	0.711	0.812	1.041
**MRPS25**	0.257	0.702	0.884	*0.006*	0.305	0.758	*0.005*	0.019	1.338	0.202	0.334	1.154
**NDUFS2**	0.892	0.975	0.992	0.699	0.953	1.031	0.717	0.820	1.024	0.343	0.491	1.072
**PPRC1**	*0.028*	0.325	1.337	0.541	0.911	1.163	*0.003*	0.013	1.747	*0.018*	0.051	1.530
**RAD23A**	0.409	0.801	0.858	*0.012*	0.379	1.907	*0.055*	0.132	0.631	0.089	0.181	1.415
**REPIN1**	0.276	0.716	1.116	0.867	0.982	0.976	*0.001*	0.007	1.399	0.112	0.216	1.232
**SDR39U1**	0.390	0.787	0.886	*0.013*	0.385	0.629	0.397	0.560	0.874	*0.004*	0.015	0.623
**SETBP1**	0.343	0.760	1.134	0.433	0.876	1.155	0.274	0.432	1.169	0.274	0.417	1.192
**SLC14A1**	0.084	0.491	0.485	0.060	0.558	1.957	*0.050*	0.123	0.616	*0.050*	0.116	2.497
**SLC18A2**	0.912	0.979	0.976	0.498	0.898	0.704	0.204	0.350	1.605	0.647	0.764	1.164
**SMC4**	0.102	0.522	0.824	0.083	0.606	0.803	*0.000*	0.001	0.630	*0.000*	0.001	0.618
**SPARC**	0.892	0.975	0.968	*0.024*	0.443	1.788	0.056	0.134	0.612	0.606	0.731	1.135
**SQLE**	*0.000*	0.004	1.661	0.145	0.693	0.774	*0.000*	0.002	1.507	*0.023*	0.062	0.705
**STRIP1**	0.380	0.780	0.943	0.560	0.917	0.947	0.074	0.165	1.147	0.055	0.124	1.160
**STX12**	0.471	0.835	0.946	0.551	0.914	1.060	0.863	0.916	1.012	0.148	0.267	1.140
**UNC45A**	*0.045*	0.390	1.165	0.212	0.753	1.184	0.977	0.987	1.003	0.760	0.845	1.028
**XPC**	0.288	0.722	0.899	0.131	0.677	1.186	*0.001*	0.003	1.407	*0.000*	0.000	1.869
