# Supplementary material for: Comparative Blood-Based Transcriptomic Profiles of Prostate Cancer Patients from South Africa and the USA: A Cross-Sectional Pilot Study
Source: J Cancer. 2026 Jan 14;17(2):382–94. doi: 10.7150/jca.126397 (PMC12825430; doi:10.7150/jca.126397)

SUPPLEMENTARY DATA

Comparative Blood-Based Transcriptomic Profiles of Prostate Cancer Patients from South Africa and the USA: A Cross-Sectional Pilot Study

Srinivas V Koduru[1*], Mark Kidd[1], Ané Pieters[2], S E Nagel[2], Robert P Millar[2] and Abdel B Halim[1]

[1]Wren Laboratories LLC, Branford CT 06405, USA

[2]Faculty of Health Sciences, University of Pretoria, Pretoria, South Africa

***Corresponding Author:**

Srinivas V Koduru, PhD

Director, Transcriptomics

Wren Laboratories LLC

Branford CT, USA

Email: skoduru@wrenlaboratories.com

Phone: +1 (475)221-8056

Supplementary Tables

Supplementary Table 1. Peripheral blood evaluation of top 10 tissue-expressed DEGs between Caucasian and Black men

Upregulated (Black PCa tissue)	PCa blood samples			Control blood samples	
	B:W fold-change	*p*-value	Same	B:W fold-change	*p*-value
ANPEP	-3.07	2.57E-09	No	-3.70	7.71E-13
IGKV2D-29	+4.39	1.04E-03	Yes	+2.76	7.72E-05
Down-regulated (Black PCa tissue)					
ANKRD36B	+1.97	1.03E-05	No	+1.48	1.87E-03
RPS24	+1.80	8.09E-15	No	+1.78	1.03E-08
SEC11C	+1.79	1.02E-08	No	+2.05	6.78E-07
SH3KBP1	-1.26	1.16E-03	Yes	-1.46	2.57E-04
STEAP4	-1.44	6.47E-03	Yes	-1.72	3.98E-03

Supplementary Table 2. Peripheral blood evaluation of top 10 tissue-expressed TFs between Caucasian and Black men

Activated (Black PCa tissue)	PCa blood samples			Control blood samples	
	B:W fold-change	*p*-value	Same	B:W fold-change	*p*-value
CTCF	-1.27	7.52E-03	No	-1.52	8.97E-07
EP300	-2.19	2.24E-11	No	-2.99	2.23E-15
ESR1	ND			**-1.85**	9.16E-03
NR3C1	-1.38	1.47E-04	No	-1.54	3.36E-04
SMARCA4	-1.68	2.75E-05	No	-1.69	9.02E-05

ND = no difference between samples

Supplementary Table 3. Peripheral blood evaluation of candidate genes associated with racial differences between Caucasian and Black men

Family	Gene	PCa blood samples		Control blood samples	
		B:W fold-change	*p*-value	B:W fold-change	*p*-value
CYP3A – testosterone regulation	CYP3A5	1.78	1.52E-03	ND	
HSD17B - Estrogen & androgen regulation	HSD17B8	1.93	3.98E-02	3.44	1.51E-04
	HSD17B10	1.42	1.72E-05	1.55	6.49E-04
	HSD17B11	1.34	3.31E-03		
Growth factor	EPHB1	-1.56	2.50E-03	-1.73	7.89E-03
BCL - apoptosis	BCL2	-1.49	1.99E-03	ND	
	**BCL3**	ND		-1.51	**8.49E-04**
	BCL6	-1.52	7.01E-03	-2.86	3.72E-07
	BCL9	-1.91	8.75E-05	ND	

ND = no difference between samples

Supplementary Table 4. Peripheral blood evaluation of candidate genes associated with racial differences between Caucasian and Black men

Family	Gene	PCa blood samples		Control blood samples	
		B:W fold-change	*p*-value	B:W fold-change	*p*-value
Fatty Acid Metabolism	*CPT1B*	-1.93	4.38E-03	*ND*	
	CPT2	-1.27	2.87E-02	-1.35	8.30E-03
	FABP5	**+2.15**	**1.61E-02**	**+3.02**	**1.58E-04**
	FTO	-1.36	1.03E-02	*ND*	
	LPCAT1	-1.29	7.99E-03	-1.42	9.72E-03

	LPCAT2	-1.62	1.98E-04	-1.95	5.58E-04
	LPCAT3	*ND*		-1.50	5.68E-03
	LPL	**+2.24**	**4.28E-02**	**+2.98**	**9.30E-04**
	PPARA	-1.44	5.08E-05	-1.35	8.14E-03
	PPARD	-1.49	3.60E-03	-1.49	1.59E-03
	PPARGC1B	-1.73	1.07E-04	-1.43	3.79E-02
	SCD	-1.87	8.91E-03	-1.53	2.49E-02
	NFKB1	-1.33	4.73E-04	-1.50	1.09E-04
	NFKB2	-1.72	3.14E-06	-1.76	4.29E-05
NFK1B	*NFKBID*	-1.31	3.13E-02	*ND*	
	NFKBIL1	**+1.50**	**4.52E-03**	**+1.44**	**2.65E-02**
	NFKBIZ	-1.41	7.05E-03	-1.43	5.06E-03
	IL10RB	-1.51	3.80E-04	-2.14	7.54E-09
	IL11RA	-1.89	6.38E-03	*ND*	
	IL13RA1	-1.26	4.12E-02	-1.81	6.78E-03
	IL15	*ND*		**+1.34**	**3.85E-02**
	IL16	-1.13	4.69E-02	-1.41	2.28E-03
	IL17RA	-1.61	1.16E-03	-2.46	3.71E-08
	IL18	*ND*		-1.44	2.56E-02
	IL18BP	-1.49	3.10E-07	*ND*	
	IL18RAP	**+2.22**	**1.25E-02**	*ND*	
	IL1R1	-1.69	3.98E-03	-2.01	1.10E-02
	IL1RL1	-2.46	8.09E-03	-3.11	2.76E-03
	IL2RA	-1.42	2.23E-02	*ND*	
	IL2RB	-1.87	3.26E-03	*ND*	
	IL23A	*ND*		**+1.98**	**2.55E-03**
	IL31RA	*ND*		-4.30	4.23E-04
	IL32	**+1.93**	**3.90E-07**	**+2.68**	**2.48E-08**
	IL4R	*ND*		-1.38	2.17E-02
	IL5RA	-1.53	1.56E-02	-2.23	7.67E-03
	IL6R	-1.42	3.62E-03	-1.92	8.01E-07
Interleukins	***IL7R***	**+1.39**	**4.03E-02**	*ND*	

Chemokine signaling	***CCL28***	*ND*		**+1.46**	**1.53E-02**
	CCL3L1	*ND*		**+2.46**	**4.16E-02**
	CCL4	**+1.67**	**1.14E-02**	**+1.85**	**1.10E-03**
	CCL4L2	**+3.68**	**2.28E-07**	**+3.22**	**2.14E-05**
	CCL5	**+1.71**	**4.78E-05**	**+1.43**	**1.24E-02**
Innate immune system	*NLRP1*	-2.05	8.78E-12	-2.16	5.92E-07
	NLRP12	*ND*		-1.93	1.52E-06
	NLRP2	*ND*		-2.46	1.32E-04
	NLRP3	-2.05	1.14E-04	-1.86	2.69E-05
	NLRP6	-1.47	1.06E-02	-2.43	1.47E-03
COX2	PTGS2	-2.44	1.37E-05	-1.77	1.10E-03
Regulation of Cytokine signaling	***SOCS2***			**+1.37**	**4.59E-02**
	SOCS4	-1.39	8.12E-03		
	SOCS6	-1.62	8.55E-03	-1.45	4.60E-02
	SOCS7	-1.47	3.89E-05		

ND = no difference between samples

Supplementary Figures and Legends

Supplementary Figure 1. GO analysis of DEGs in RSA-PCa vs USA-PCa.

Supplementary Figure 2. GO analysis of DEGs in RSA-Controls vs USA-Controls.

Supplementary Figure 3. Gene expression analysis: PCa vs. controls.
3A. Venn diagram showing common genes between RSA and USA population, without statistical filtering, where show. More 90% of the genes were common to both groups.
3B. Venn diagram showing commonly expressed genes after applying a fold change (FC) >2 and p< 0.05 filter. Only <10% of genes were commonly expressed.

Supplementary Figure 4.
4A. Hierarchal clustering (heatmap) showing the clustering of gene expression profiles in blood samples from RSA-PCa vs RSA-controls.
4B. Circos plot identifying statistically significant gene expression in RSA-PCa vs RSA-Controls. Chromosome hotspots identified in Chromosome 1, 15, 16 and 17.
4C. Volcano plot displaying the significantly differentially expressed genes (p<0.05) between RSA-PCa vs RSA-Control populations.
4D. Major pathways regulating ribosomal and hematopoietic cell lineages enriched in RSA-PCa samples.

Supplementary Figure 5.
5A. Hierarchal clustering (heatmap) showing the clustering of gene expression profiles in blood samples from USA-PCa vs USA-controls.
5B. Circos plot identifying statistically significant gene expression in USA-PCa vs USA-Controls. Chromosome hotspots identified in Chromosomes 1, 5, 6, 15 and 19.
5C. Volcano plot displaying the significantly differentially expressed genes (p<0.05) between USA-PCa vs USA-Control populations.
5D. Pathways significantly enriched in USA-PCa samples include protein export and endoplasmic reticulum pathways.

Supplementary Figure 1

RSA-PCa vs USA-PCa

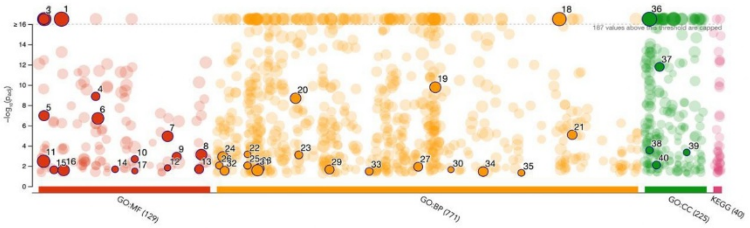

ID	Source	Term ID	Term Name	p_{adj} (query_1)
1	GO:MF	GO:0005515	protein binding	3.852×10^{-153}
2	GO:MF	GO:0003824	catalytic activity	9.449×10^{-27}
3	GO:MF	GO:0003712	transcription coregulator activity	5.698×10^{-20}
4	GO:MF	GO:0019843	rRNA binding	1.329×10^{-9}
5	GO:MF	GO:0003729	mRNA binding	1.075×10^{-7}
6	GO:MF	GO:0030234	enzyme regulator activity	1.978×10^{-7}
7	GO:MF	GO:0060090	molecular adaptor activity	1.159×10^{-5}
8	GO:MF	GO:0140657	ATP-dependent activity	7.438×10^{-4}
9	GO:MF	GO:0090079	translation regulator activity, nucleic acid bindi...	1.216×10^{-3}
10	GO:MF	GO:0046933	proton-transporting ATP synthase activity, rota...	2.069×10^{-3}
11	GO:MF	GO:0003677	DNA binding	3.386×10^{-3}
12	GO:MF	GO:0055105	ubiquitin-protein transferase inhibitor activity	1.507×10^{-2}
13	GO:MF	GO:0140104	molecular carrier activity	1.993×10^{-2}
14	GO:MF	GO:0034450	ubiquitin-ubiquitin ligase activity	2.050×10^{-2}
15	GO:MF	GO:0004715	non-membrane spanning protein tyrosine kina...	2.363×10^{-2}
16	GO:MF	GO:0008270	zinc ion binding	2.563×10^{-2}
17	GO:MF	GO:0046934	1-phosphatidylinositol-4,5-bisphosphate 3-kin...	3.072×10^{-2}
18	GO:BP	GO:1901564	organonitrogen compound metabolic process	2.357×10^{-106}
19	GO:BP	GO:0051301	cell division	1.662×10^{-10}
20	GO:BP	GO:0016032	viral process	2.018×10^{-9}
21	GO:BP	GO:1902600	proton transmembrane transport	8.166×10^{-6}
22	GO:BP	GO:0006122	mitochondrial electron transport, ubiquinol to c...	6.934×10^{-4}
23	GO:BP	GO:0017004	cytochrome complex assembly	7.846×10^{-4}
24	GO:BP	GO:0001701	in utero embryonic development	1.214×10^{-3}
25	GO:BP	GO:0006123	mitochondrial electron transport, cytochrome ...	8.900×10^{-3}
26	GO:BP	GO:0000470	maturation of LSU-rRNA	8.900×10^{-3}
27	GO:BP	GO:0048144	fibroblast proliferation	1.169×10^{-2}
28	GO:BP	GO:0007163	establishment or maintenance of cell polarity	1.721×10^{-2}
29	GO:BP	GO:0031123	RNA 3'-end processing	2.193×10^{-2}
30	GO:BP	GO:0060397	growth hormone receptor signaling pathway vi...	2.232×10^{-2}
31	GO:BP	GO:0007017	microtubule-based process	2.625×10^{-2}
32	GO:BP	GO:0001841	neural tube formation	2.949×10^{-2}
33	GO:BP	GO:0036260	RNA capping	3.363×10^{-2}
34	GO:BP	GO:0071383	cellular response to steroid hormone stimulus	3.503×10^{-2}
35	GO:BP	GO:0099116	tRNA 5'-end processing	4.675×10^{-2}
36	GO:CC	GO:0005737	cytoplasm	1.151×10^{-211}
37	GO:CC	GO:0030684	preribosome	1.666×10^{-12}
38	GO:CC	GO:0005732	sno(s)RNA-containing ribonucleoprotein compl...	2.753×10^{-4}
39	GO:CC	GO:0071564	npBAF complex	4.570×10^{-4}
40	GO:CC	GO:0016592	mediator complex	7.928×10^{-3}

**version**	e110_eg57_p18_51f3c30
**date**	9/27/2023, 3:07:17 PM
**organism**	hsapiens

g:Profiler

Supplementary Figure 2

RSA-Controls vs USA-Controls

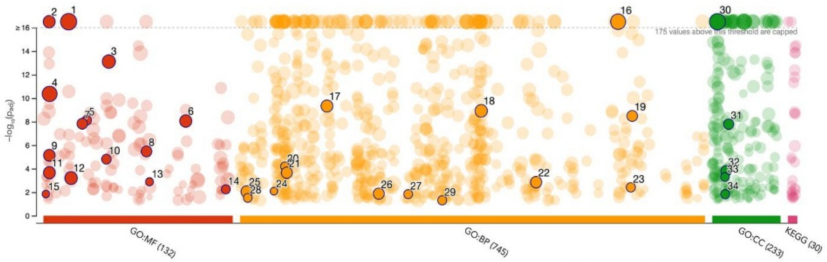

ID	Source	Term ID	Term Name	p_{adj} (query_1)
1	GO:MF	GO:0005515	protein binding	3.205×10^{-131}
2	GO:MF	GO:0003712	transcription coregulator activity	4.681×10^{-26}
3	GO:MF	GO:0030234	enzyme regulator activity	7.722×10^{-14}
4	GO:MF	GO:0003824	catalytic activity	4.324×10^{-11}
5	GO:MF	GO:0015453	oxidoreduction-driven active transmembrane t...	7.702×10^{-9}
6	GO:MF	GO:0060090	molecular adaptor activity	8.798×10^{-9}
7	GO:MF	GO:0015078	proton transmembrane transporter activity	1.503×10^{-8}
8	GO:MF	GO:0045182	translation regulator activity	3.380×10^{-6}
9	GO:MF	GO:0003729	mRNA binding	7.300×10^{-6}
10	GO:MF	GO:0019843	rRNA binding	1.559×10^{-5}
11	GO:MF	GO:0003735	structural constituent of ribosome	2.173×10^{-4}
12	GO:MF	GO:0008270	zinc ion binding	6.198×10^{-4}
13	GO:MF	GO:0046933	proton-transporting ATP synthase activity, rota...	1.291×10^{-3}
14	GO:MF	GO:0140938	histone H3 methyltransferase activity	5.687×10^{-3}
15	GO:MF	GO:0001054	RNA polymerase I activity	1.422×10^{-2}
16	GO:BP	GO:1901564	organonitrogen compound metabolic process	6.207×10^{-85}
17	GO:BP	GO:0016032	viral process	4.710×10^{-10}
18	GO:BP	GO:0051301	cell division	1.203×10^{-9}
19	GO:BP	GO:1902600	proton transmembrane transport	3.272×10^{-9}
20	GO:BP	GO:0007020	microtubule nucleation	6.517×10^{-5}
21	GO:BP	GO:0007163	establishment or maintenance of cell polarity	2.205×10^{-4}
22	GO:BP	GO:0071559	response to transforming growth factor beta	1.402×10^{-3}
23	GO:BP	GO:1902459	positive regulation of stem cell population mai...	3.856×10^{-3}
24	GO:BP	GO:0006122	mitochondrial electron transport, ubiquinol to c...	8.176×10^{-3}
25	GO:BP	GO:0001701	in utero embryonic development	8.928×10^{-3}
26	GO:BP	GO:0032869	cellular response to insulin stimulus	1.327×10^{-2}
27	GO:BP	GO:0036260	RNA capping	1.446×10^{-2}
28	GO:BP	GO:0001782	B cell homeostasis	3.029×10^{-2}
29	GO:BP	GO:0045454	cell redox homeostasis	4.933×10^{-2}
30	GO:CC	GO:0005737	cytoplasm	5.541×10^{-188}
31	GO:CC	GO:0030684	preribosome	1.643×10^{-8}
32	GO:CC	GO:0019814	immunoglobulin complex	1.564×10^{-4}
33	GO:CC	GO:0016514	SWI/SNF complex	5.098×10^{-4}
34	GO:CC	GO:0016592	mediator complex	1.467×10^{-2}

**version**	e110_eg57_p18_51f3c30
**date**	9/27/2023, 4:55:18 PM
**organism**	hsapiens

g:Profiler

Supplementary Figure 3 PCa vs Controls

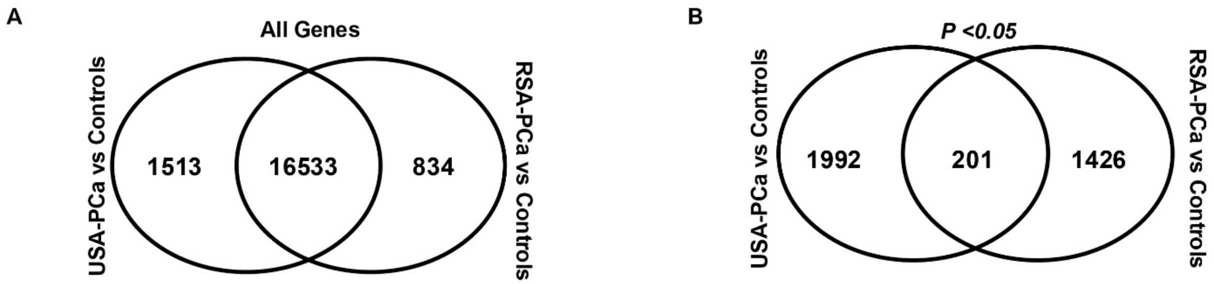

Supplementary Figure 4 RSA-PCa vs Controls

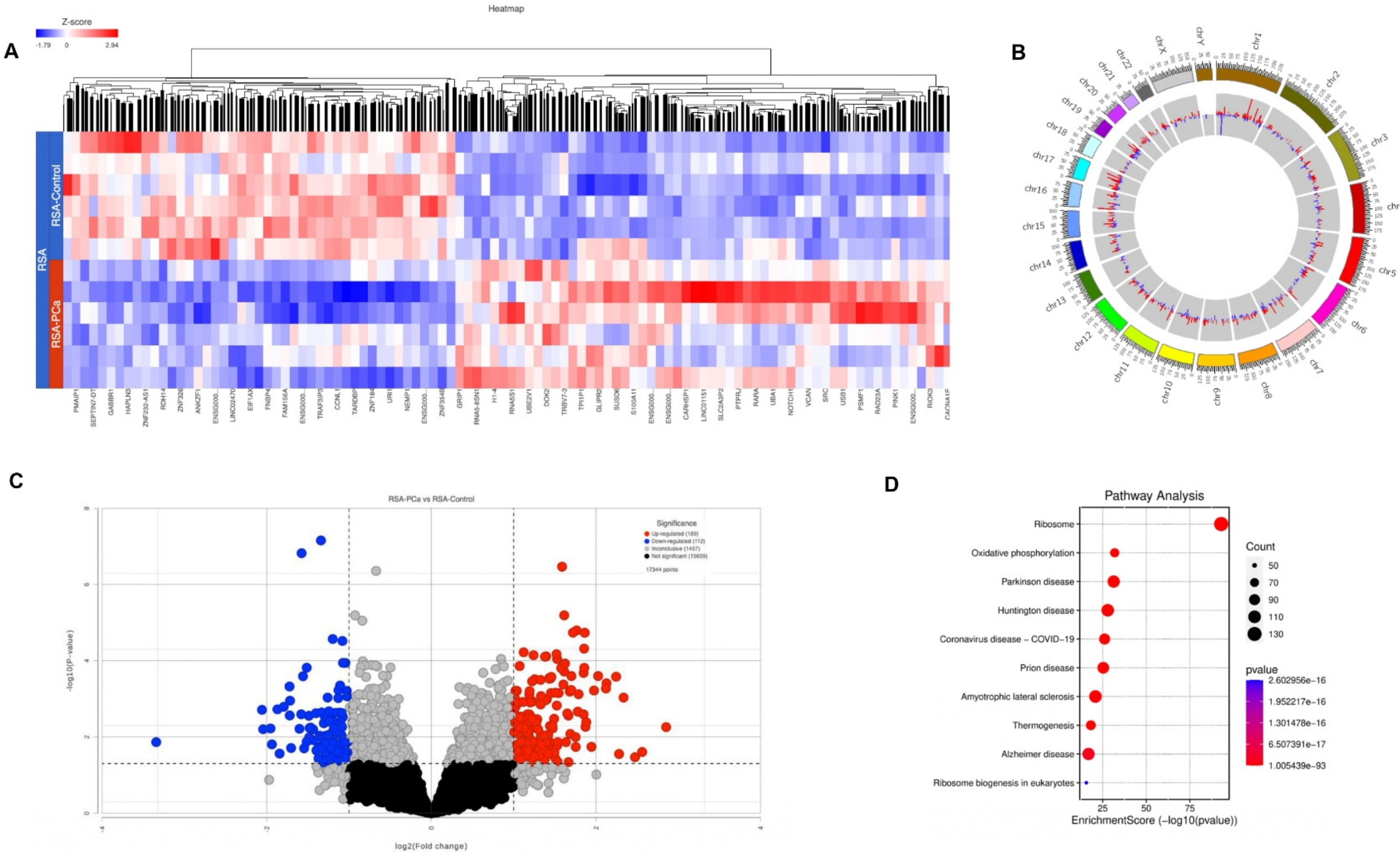

Supplementary Figure 5 USA-PCa vs Controls

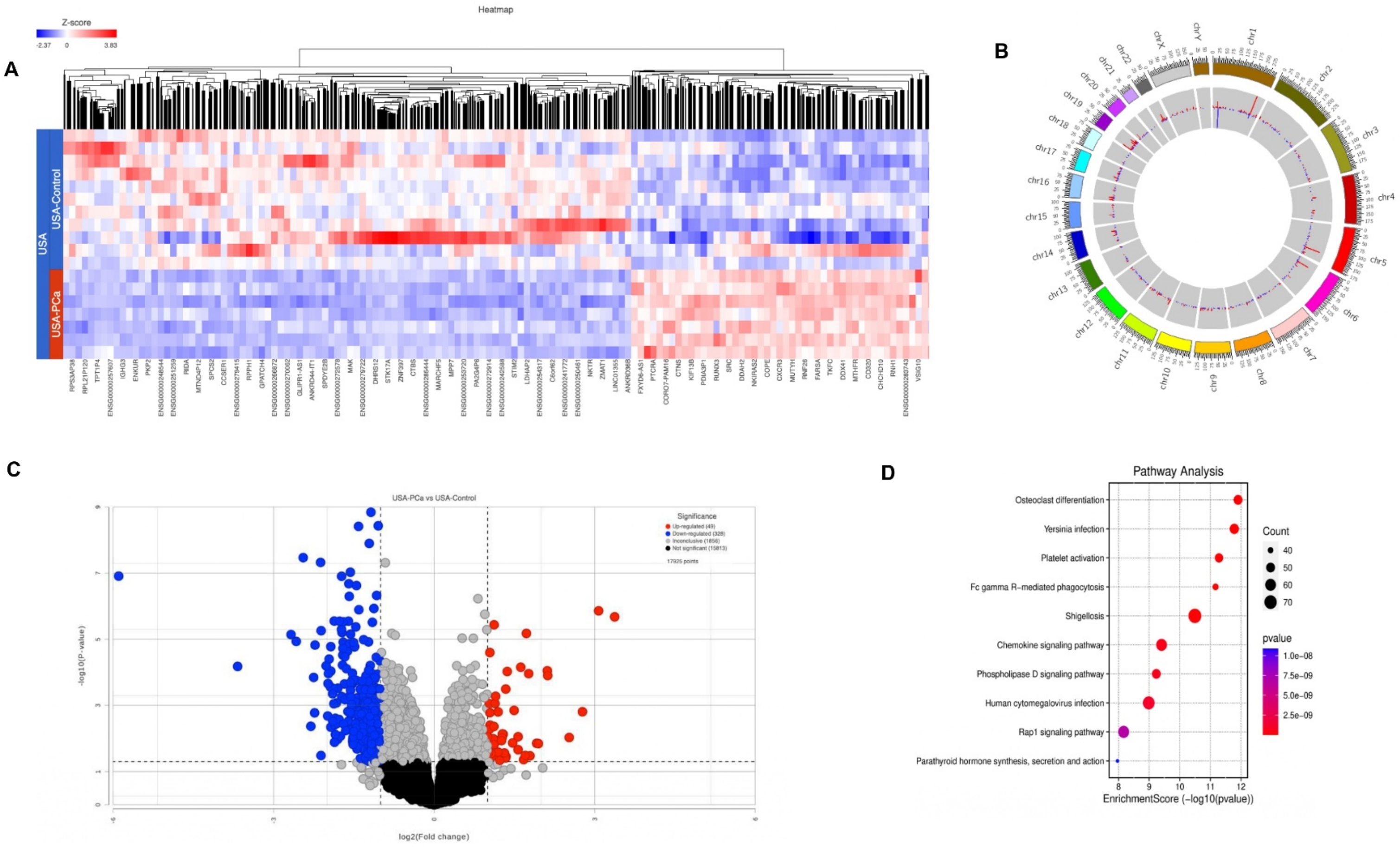

Supplement: Supplementary file 1 — Supplementary figures and tables. [file jcav17p0382s1.pdf]
